# Should Employers Invest in Employability? Examining Employability as a Mediator in the HRM – Commitment Relationship

**DOI:** 10.3389/fpsyg.2019.00717

**Published:** 2019-04-11

**Authors:** Jos Akkermans, Maria Tims, Susanne Beijer, Nele De Cuyper

**Affiliations:** ^1^School of Business and Economics, Vrije Universiteit Amsterdam, Amsterdam, Netherlands; ^2^Research Group Work, Organization, and Personnel Psychology, KU Leuven, Leuven, Belgium

**Keywords:** employability, HR practices, workplace commitment, social exchange, career

## Abstract

This study investigates the relationship between perceived investments in Human Resource (HR) practices and workplace commitment, from the perspective of social exchange theory. An innovative feature is that we introduce perceived employability as a potential mediator, thus bringing in a career perspective: our argument is that perceived investments in HR practices promote feelings of employability, which then create workplace commitment. Based on a 6-week follow-up sample (*N* = 437) and a 1-year follow-up sample (*N* = 127), the results of structural equation modeling analyses mostly provided support for our hypotheses. Participation and communication practices were linked to commitment via employability (in both samples), and training and development only in the short term (6-week sample). Performance feedback and reward practices, however, were unrelated to commitment via employability. Overall, our findings show that employees bring in career considerations, employability concerns in particular, in the exchange with their employer. In addition, we contribute to filling the HRM “black box” by showing that employability might be an explanatory mechanism in the HR practices – outcome relationship.

## Introduction

The relationship between Human Resource (HR) investments, (i.e., HR practices) and commitment is well-established (Bowen and Ostroff, 2004; Kehoe and Wright, 2013), often supported with social exchange dynamics as underlying principle: when employees perceive investments from the organization, they feel a need to reciprocate in the form of commitment. What is often neglected, though, is that social exchange is aimed at a mutual win. We believe that employability presents such a win. Perceived employability concerns employees’ perceptions of their chance on employment in the internal and/or the external labor market (Forrier et al., 2009; Vanhercke et al., 2014), and has been advanced as an asset for employees in current times of ongoing change (Van Der Heijde and Van Der Heijden, 2006). Thus, the win for employees is that it provides them with a form of employment security. Employability could also be a key asset for organizations, in particular when employees see the connection between the organization’s HRM investments and their own employability. This connection could foster workplace commitment (Klein et al., 2012), thereby fulfilling the organization’s need for a committed workforce with up-to-date knowledge and skills (Conway, 2004; Nilsson and Ellström, 2012).

Although the value of employability enhancement is clear for individuals and organizations alike, much less is known about whether employability can be enhanced through HR practices (cf. Nelissen et al., 2017). One key mechanism that may provide a better understanding of this process is social exchange (Cropanzano and Mitchell, 2005): when employees perceive HRM investments in the form of HR practices that can enhance their employability, this may create a mutual win through a social exchange process. First, these practices will help employees to develop valuable knowledge and skills and second, these practices will benefit the employer as employees will value these investments and will show commitment in return.

Our aim is to probe the potential mediating role of employability in the perceived HR practices– employee workplace commitment relationship in greater detail using a social exchange perspective. Although the selection of perceived HR practices (which we will refer to as “HR practices” in the remainder of this paper) in empirical research differs across studies, three practices are consistently studied: (1) training and development, (2) employee participation and communication, and (3) performance feedback and reward (Gould-Williams, 2007; Chew and Chan, 2008). We therefore use these three practices in our study. Employability has been approached in different ways: our focus is upon employee perceptions of their chances in the internal, (i.e., perceived internal employability) and external, (i.e., perceived external employability) labor market (Vanhercke et al., 2014). Perceived employability was chosen because it fits a social exchange perspective: employees will only reciprocate when they themselves perceive a win in terms of increased employability.

Our study makes four contributions to the literature. The first contribution is that our study brings in a career perspective to Social Exchange (SE) Theory. This theory has often been applied from the perspective of tangible rewards (e.g., financial incentives or promised promotions) yet we argue that there is also a “social career exchange” in which reciprocity is activated because of long-term gains for both parties. Second, our study contributes to filling the so-called “black box” of HRM (Becker and Huselid, 2006) by studying whether employability might be a mechanism through which HR practices can bring about enhanced workplace commitment. As a third contribution, we study the relationship between HR practices and employability, which is an important but understudied area (Van Harten et al., 2017). Doing so can provide a better understanding of the relational nature of employability – that is: employability is not fully under control of the individual but always part of an interdependent relationship – which has been brought forward as a blind spot in employability research (Forrier et al., 2018). Finally, research on the employability – commitment relationship has been mixed thus far with some studies arguing that high levels of perceived employability decrease commitment while others show no or a positive relationship (for a review, see Philippaers et al., 2017). Bringing in a SE perspective (Cropanzano and Mitchell, 2005), we argue that employer investments are the key to initiate reciprocity between mutually dependent parties in the employment relationship. The implication is that enhanced employability can go hand in hand with high levels of commitment (cf. Philippaers et al., 2017).

### HR Practices, Commitment and Employability: A Social Exchange Perspective

Social Exchange theory (Cropanzano and Mitchell, 2005) is based on the premise that social exchanges in the workplace generate mutual obligations between employers and employees, in which the actions of one party are contingent upon the actions of another (Blau, 1964). At the core of social exchange is the norm of reciprocity (Cropanzano and Mitchell, 2005), which states that investments from one party are reciprocated by the other party (Gouldner, 1960). Applied to this study, employee perceptions of organizational investments in the form of HR practices should be reciprocated by employees by investing in the organization, for example through commitment. Workplace commitment was conceptualized by Klein et al. (2012) as “a volitional psychological bond reflecting dedication to and responsibility for a particular target” (p. 137). This particular conceptualization of commitment means that it can be targeted at various aspects in peoples’ work, such as the team or department they are part of, or the organization in general. Workplace commitment is a crucial outcome for organizations, as they benefit from workers that are psychologically committed (Van Rossenberg et al., 2018). In this study, we used the conceptualization of workplace commitment of Klein et al. (2014) rather than the often used conceptualization of affective, normative, and continuance commitment (Allen and Meyer, 1990; Meyer et al., 2002). The reason is that we are interested in a general sense of commitment that employees have toward their employer as a result of investments in their employability. To explain further, Klein et al. (2012) argue that a target free approach of commitment reflects a volitional dedication and responsibility, and is applicable to any workplace setting. Given our research focus on how social exchange processes might explain the link between HR practices, employability, and commitment, it is especially this volitional dedication and felt responsibility toward the employer that we want to tap into.

As mentioned previously, one way to stimulate employees’ commitment is to invest in them via HR practices. In line with prior research and because of their regular use in HRM research, we focus specifically on the HR practices of training and development, participation and communication, and performance feedback and reward (Gould-Williams, 2007; Chew and Chan, 2008). Furthermore, we assessed the perceptions of HR practices among employees, which has become a common method for measuring HR practices in recent years (Beijer et al., 2019). Specifically, we focused on employees’ *evaluations* of these practices, which is most likely to tap into attitudinal and affective outcomes, such as commitment (Beijer et al., 2019).

Studies in the realm of HRM research typically argue that HRM investments are appraised positively by employees, and this positive perception of their employer will lead to trust and long-term commitment (Bowen and Ostroff, 2004; Kehoe and Wright, 2013; Van Harten et al., 2016). Thus, based on social exchange mechanisms, it is likely that investing in employees’ development, creating clear and open communication, and providing them with feedback and fair rewards will show employees that the organization cares about them and invests in their development. This should trigger the norm of reciprocity and lead employees to respond with enhanced workplace commitment (Whitener, 2001; Tremblay et al., 2010; De Cuyper and De Witte, 2011).

Hypothesis 1: *HR practices will be positively related to workplace commitment*.

Commitment has particular resonance in tandem with HR practices and employability. Employability can be characterized as the individual’s chance of finding a job on the internal and/or external labor market (Forrier and Sels, 2003), and research on this topic has flourished in recent years (Akkermans and Kubasch, 2017). The scholarly discussion has mostly focused on input-based and outcome-based approaches to employability and their connection. Employability competencies (Van Der Heijde and Van Der Heijden, 2006) and dispositions (Fugate et al., 2004) are input to achieve high levels of perceived employability (Forrier et al., 2015). Thus, whereas input-based approaches focus on aspects that can enhance the chance of finding a job, outcome-based approaches directly assess employment opportunities, for example via employee perceptions (Forrier et al., 2015). Our focus here is specifically on those perceptions of employability, as we want to study whether HR practices can enhance employees’ perceived opportunities of finding alternative employment internally and externally.

There is a lively debate on whether employer investments in employability would lead to higher vs. lower levels of commitment among employees. This debate is often referred to as the employability management paradox (cf. De Cuyper and De Witte, 2011; Philippaers et al., 2016, 2017). Some authors say that employers should invest in employability as this provides a competitive advantage (Van Harten et al., 2016), while others highlight potential risks of employability investments, most notably in terms of turnover (Benson, 2006; De Cuyper et al., 2011).

We question this paradox: it rather artificially separates employee and employer wins, while employability may in fact create a mutual win. Viewed from a social exchange perspective, employees are likely to perceive organizational investments in their employability as a sign of being valued by their employer and of how committed the organization is to them (Blau, 1964; Eisenberger et al., 1990; Wayne et al., 1997). This would activate the norm of reciprocity based on creation of mutual trust, thus resulting in a more committed workforce (Lavelle et al., 2007). If so, this implies that perceived employability may mediate the relationship between HR practices and workplace commitment. This means that investments on the part of the employer bring about a personal career-related win for the employee, because the investment that their employer makes for them can help them develop important knowledge and skills, thereby potentially enhancing work and career success (Akkermans and Tims, 2017).

It is important to recognize that perceived employability is shaped by factors tied to the person (e.g., education; Berntson et al., 2006) but also by factors in the larger environment, for example by investments on the part of the employer (Van Der Heijden and Bakker, 2011; Vanhercke et al., 2014; Nelissen et al., 2017). Applied to this study, this indicates that perceived employability can be shaped by organizational HRM investments. Such investments from the employer create a need for the employee to reciprocate and feeds forward to beneficial outcomes such as workplace commitment (Foa and Foa, 1980; Van Harten et al., 2016). The win for employers then is twofold: HRM investments result in an employable workforce with up-to-date knowledge and skills, and also a committed workforce as a result of those investments. In all, the mutual win of HRM investments in employability is likely to bind both parties, thus promoting workplace commitment. This leads to the general idea that employability explains the relationship between HR practices and commitment.

Prior research has indeed pointed in this direction (e.g., Conway and Monks, 2009; De Vos et al., 2011), yet has not differentiated between perceived internal and external employability. The HR practice – employability – commitment dynamics are plausible for internal employability but may be less plausible for external employability: HRM investments can be organization-specific and thus primarily aimed at enhancing internal employability (cf. Kraaijenbrink et al., 2010). We would argue, however, that the boundaries between the internal and external labor market are not that strict, meaning that an investment in peoples’ internal employability would likely also strengthen their external opportunities (cf. Guest, 1987). For example, even when employees participate in organization-specific projects, their participation in itself provides them with knowledge and skills that can be relevant to other organizations. As a result, although these investments are internally oriented, they do present a personal win for the employee in the form of increased marketability (Shore et al., 2006). This personal win could trigger the need to reciprocate because employees may want to safeguard this win also in the future, and provoking employer investment may be a means to do so.

To conclude, HR practices are likely positively related to commitment via both forms of perceived employability, albeit in a slightly different social exchange process. For internal employability, this social exchange centers around employees needing to reciprocate the investments their employer makes in their development as it is a signal that their employer values them. For external employability, employees reciprocate HR practices aimed at their development with increased commitment, because it allows them a career-related personal win of continuous development opportunities, and they want to safeguard those opportunities in the future. Evidence supports this idea: employees are more committed to the organization when provided the opportunity to develop marketable skills and experience (Galunic and Anderson, 2000; Craig et al., 2002; Van Harten et al., 2016).

Hypothesis 2: *Perceived internal employability will mediate the positive relationship between HR practices and workplace commitment.*

Hypothesis 3: *Perceived external employability will mediate the positive relationship between HR practices and workplace commitment.*

## Materials and Methods

### Procedure and Participants

A diverse sampling strategy using social media networks (e.g., LinkedIn) was used to increase the heterogeneity of the participants, which facilitates generalizability of the findings (Demerouti and Rispens, 2014). The study consisted of three measurement waves: The predictor variables were assessed at T1, and the mediator and outcome variables were assessed at T2 and T3. In total, 1,003 respondents participated in the first measurement wave (T1). After 6 weeks, the second measurement wave (T2) resulted in complete data of 437 employees who participated at both T1 and T2. A year after the first measurement wave, we invited all T1 respondents to fill in the third survey (T3). We received completed surveys from 127 respondents. Due to the small sample size at T3 and the low number of matched employees at all measurement occasions (*N* = 89), we decided to analyze the data as if there were two samples: a 6-week follow-up T1-T2 sample (*N* = 437) and a one-year follow-up T1-T3 sample (*N* = 127).

The added benefit of this approach is that it offers us both a short-term and a long-term measurement of the study variables. In the T1-T2 sample, the mean age of the respondents was 34.55 years (*SD* = 12.31) and the majority was female (60.6%). On average, participants worked 32.32 h per week. In the T1-T3 sample, the average age of the respondents was 36.42 years (*SD* = 12.19), the majority was female (66.4%) and participants worked for 33.61 h per week (*SD* = 9.08). We assessed whether dropouts differed from participants on the demographical data. Results showed that dropouts from T1 to T2 were more likely to be male and shorter tenured employees (*t* = -2.23, *p* < 0.01 and *t* = -2.18, *p* < 0.01, respectively). Dropouts between T1 and T3 were also more likely to be male (*t* = -2.88, *p* < 0.01) but did not differ on other demographics.

With regard to research ethics, we did not seek approval from an ethical committee as the survey research that we performed was exempt from such approval in the country in which the study was performed, (i.e., Netherlands) and by the institutions leading this project. All research participants were informed in an introductory explanation of the survey that they would formally agree to participate in the research by filling out the survey, thereby giving informed consent if they chose to participate. All participants were informed that their participation was completely voluntary and that they could quit at any time. Thus, even though formal ethical approval was not required, we did ensure an ethical research process.

### Measurement Instruments

*Perceptions of HR practices* were measured with a 5-point Likert scale ranging from 1 (*completely disagree*) to 5 (*completely agree*), and were based on Beijer (2014). Training and development practices were measured with six items, for example: “Employees in this organization are provided with sufficient opportunities for training and development” (α_T1_ = 0.86). For participation and communication practices, five items were used, an example being: “Employees in this organization are often asked by their supervisor to participate in decisions” (α_T1_ = 0.88). Performance feedback and reward practices were measured using four items, for example: “Employees in this organization receive regular and constructive feedback on how well they do their job” (α_T1_ = 0.82).

*Perceived employability* was measured with eight items from De Cuyper and De Witte (2008), which were slightly adapted by Akkermans et al. (2013) for a Dutch population. Items were answered using a 5-point Likert scale ranging from 1 (*completely disagree*) to 5 (*completely agree*). Perceived internal employability was measured using four items, for example: “I am able to proceed into other jobs with my current employer (α_T2_ = 0.86, α_T3_ = 0.83). Perceived external employability was also measured using four items, such as: “I would find another job rather quickly if I would search for it” (αT2 = 0.87, αT3 = 0.83).

*Workplace commitment* was measured with four items of the unidimensional measure developed by Klein et al. (2014), on a 5-point Likert scale ranging from 1 (*not at all*) to 5 (*extremely*). An example item is “How committed are you to [target]?,” where the target was formulated as the organization (αT2 = 0.93, αT3 = 0.93).

### Strategy of Analysis

Before testing the hypothesized model, the validity of the constructs was assessed using confirmatory factor analysis (CFA). Next, we examined the hypothesized model using structural equation modeling. When fit was inadequate, modification indices were examined to identify misspecifications and where possible, to correct them. The mediating role of perceived internal and external employability in the relationship between HR practices and organizational commitment was assessed using the bootstrap option (500 bootstrap samples). In the model, we specified correlations between the three HRM practices and between perceived internal and external employability because of their theoretical similarities. Several demographic variables were included in the analyses as controls, (i.e., age, gender, tenure, working hours per week). Based on the correlation table, respondent age was the only control variable that significantly related to the dependent variables in the analyses and is therefore reported in the results.

## Results

### Descriptive Statistics, Measurement Model, and CFAs

The correlations among the study variables are presented in Table 1. Most of the correlations were in line with our expectations. Interestingly, the correlation between perceived internal employability and workplace commitment was stronger than the correlation between external perceived employability, yet both were positive.

**Table 1:** Correlation table.

	M	*SD*	1	2	3	4	5	6	7	8	9	10	11
1 Age	35.55/36.42	12.31/12.19	–	–0.04	–0.16	–0.01	0.65**	0.04	0.06	0.06	–0.29**	–0.32**	–0.00
2 Gender	1.61/1.69	0.49/0.46	–0.01	–	0.07	–0.27**	–0.01	0.04	–0.09	–0.03	–0.06	0.05	–0.17
3 Educational level	3.81/4.09	1.53/1.44	–0.18**	0.08	–	0.08	–0.01	0.00	–0.12	0.06	0.07	0.04	–0.03
4 Working hours	36.80/37.78	10.54/9.26	0.02	–0.31**	–0.05	–	–0.10	–0.03	0.10	0.14	0.16	0.05	0.22*
5 Tenure	5.92/7.01	8.09/9.03	0.18**	0.04	0.08	–0.08	–	0.15	0.06	0.03	–0.40**	–0.22*	0.01
6 HRM TD	3.32/2.98	0.80/0.47	–0.03	–0.04	0.04	0.18**	0.02	–	0.53**	0.24**	–0.07	0.11	0.11
7 HRM FR	3.21/3.14	0.77/0.84	–0.05	–0.12*	0.04	0.08	–0.02	0.54**	–	0.52**	–0.03	0.14	0.10
8 HRM PC	3.50/3.51	0.78/0.78	–0.08	–0.05	0.09	0.09	–0.07	0.44**	0.53**	–	0.17	0.26**	0.31**
9 PE Ext.	3.20/3.28	0.86/0.80	–0.37**	–0.17**	0.11*	0.17**	–0.04	0.02	0.03	0.09*	–	0.37**	0.29**
10 PE Int.	3.46/3.57	0.87/0.85	–0.30**	–0.13**	0.09*	0.16**	–0.07	0.31**	0.27**	0.25**	0.41**	–	0.38**
11 Commitment	4.73/4.83	0.87/0.94	0.13**	–0.10*	–0.05	0.25**	–0.12*	0.15**	0.17**	0.32**	0.09	0.22**	–


*Correlations below diagonal are correlations in sample 1 (T1–T2, *N* = 437), correlations above diagonal are correlations in sample 2 (T1–T3, *N* = 127). First mean and standard deviation reflect sample 1, second number reflects sample 2. TD, training and development; FR, performance feedback and reward; PC, participation and communication; PE Ext., perceived external employability; PE Int., perceived internal employability. ^∗^*p* < 0.05, ^∗∗^*p* < 0.001.*

The measurement model consisted of six latent variables, (i.e., three HR practices, perceived internal and external employability, and organizational commitment). To assess the validity of the measurement instruments, three models were compared. As shown in Table 2, in both samples, the hypothesized six-factor measurement model (model 3) showed the best fit to the data compared to the alternative models. In sample 1, model 3, the model fit was adequate, however, in sample 2, the model fit of the hypothesized model (model 3) was inadequate and modification indices suggested to add a correlation between two similar items assessing perceived external employability and between four items assessing feedback and reward practices. These correlations were added in both measurement models, resulting in improved and adequate model fit in both samples. All items loaded significantly on their respective factors (all *p*’s < 0.01).

**Table 2:** Result of confirmatory factor analyses.

	(χ^2^)	df	*p*	RMSEA	CFI	TLI	Δχ^2^/df	*p*
**Sample 1: T1–T2**								
M1	5330.42	324	0.00	0.19	0.34	0.28		
M2	2719.11	321	0.00	0.13	0.68	0.65	2611.31/3	0.00
M3	933.15	309	0.00	0.07	0.92	0.91	1785.96/12	0.00
M3 trimmed	700.91	306	0.00	0.05	0.95	0.94	232.24/3	0.00
**Sample 2: T1–T3**								
M1	1869.74	324	0.00	0.20	0.33	0.27		
M2	1119.34	321	0.00	0.14	0.65	0.62	750.40/3	0.00
M3	591.40	309	0.00	0.09	0.88	0.86	527.94/12	0.00
M3 trimmed	499.64	306	0.00	0.07	0.92	0.90	91.76/3	0.00


*M1, all items load on 1 factor. M2, predictors, mediators, and outcome load on a separate factor (3 factor model). M3, each measure loads on their individual factor (6 factor model).*

### Test of Hypotheses: Six-Week Follow-Up

The hypotheses were tested by adding all hypothesized paths to the measurement model. We then created a more parsimonious model by omitting non-significant relationships. Results are based on this more parsimonious model. The non-significant findings relevant to the hypotheses testing are derived from the full model. Analyses show that the parsimonious model fits the data well (*χ*^2^ = 509.26, *df* = 237, CFI = 0.96, TLI = 0.95, RMSEA = 0.05).

Participation and communication was related to workplace commitment (β = 0.36, *p* < 0.01), however, training and development (β = -0.07, *p* = 0.24) and feedback and reward (β = -0.05, *p* = 0.52) were not. These results provide partial support for H1.

To assess the indirect relationships (H2 and H3), we first present results on the relationships between HR practices and perceived employability. Training and development and participation and communication were positively related to perceived internal employability (β = 0.25, *p* < 0.01 and β = 0.13, *p* = 0.02, respectively). However, feedback and reward was not significantly related to perceived internal employability (β = 0.09, *p* = 0.24). Perceived internal employability was positively related to workplace commitment (β = 0.17, *p* < 0.01). Next, we found that participation and communication related to perceived external employability (β = 0.15, *p* = 0.01), whereas training and development and feedback and reward were not related to perceived external employability (β = 0.05, *p* = 0.44 and β = -0.05, *p* = 0.53, respectively). Perceived external employability was positively related to workplace commitment (β = 0.11, *p* = 0.04). The bootstrapped indirect effects from each significant HR practice to workplace commitment via perceived internal and external employability were assessed. Participation and communication (estimate = 0.04, *p* < 0.01, B-CCI: 0.01–0.09) and training and development (estimate = 0.05, *p* = 0.02, B-CCI: 0.01–0.09) were indirectly associated with commitment via perceived employability. Thus, the results provide partial support for H2 and H3.

Finally, as an additional check, we constrained the paths between HR practices and perceived internal and external employability to be equal as well as the paths from perceived internal and external employability to organizational commitment. This served to examine whether the specific relationships were different in strength. Model fit of this constrained model did not change significantly compared to the model without these constraints (Δχ^2^ = -2.30, *p* = 0.51), indicating that the relationships were not significantly different from each other.

### Test of Hypotheses: One-Year Follow-Up

Analyses show that the parsimonious model fits the data well (*χ*^2^ = 180.72, *df* = 125, CFI = 0.96, TLI = 0.95, RMSEA = 0.06). Participation and communication was related to workplace commitment (β = 0.20, *p* = 0.03), however, training and development (β = 0.14, *p* = 0.25) and feedback and reward (β = -0.19, *p* = 0.19) were not. These results provide partial support for H1. Next, to test the indirect effects (H2 and H3), we first present results on the HR practices – perceived employability relationships. Results showed that participation and communication was positively related to perceived internal employability (β = 0.30, *p* < 0.01). However, training and development, and feedback and reward were not significantly related to perceived internal employability (β = 0.13, *p* = 0.29 and β = -0.09, *p* = 0.52, respectively). Perceived internal employability was positively related to workplace commitment (β = 0.27, *p* = 0.01).

With regard to external employability, we found that participation and communication was the only HR practice that related to perceived external employability (β = 0.28, *p* < 0.01). Training and development, and feedback and reward were not related to perceived external employability (β = 0.06, *p* = 0.66 and β = -0.23, *p* = 0.14, respectively). Perceived external employability was positively related to workplace commitment (β = 0.22, *p* = 0.03). Based on these results, the bootstrapped indirect effect from participation and communication to commitment via perceived internal and external employability was assessed, which was significant (estimate = 0.15, *p* < 0.01, B-CCI: 0.04–0.28), providing partial support for H2 and H3.

**FIGURE 1:**
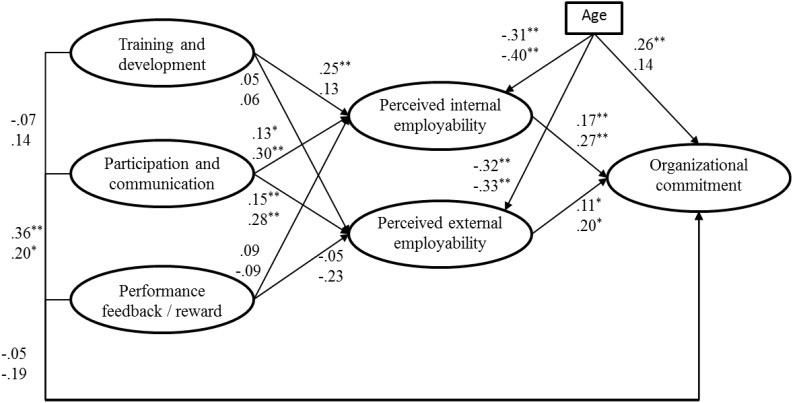
Results of structural equation modeling analyses. First value refers to sample 1, second value refers to sample 2. ^∗∗^*p* < 0.01; ^∗^*p* < 0.05.

Finally, we found that the model in which the paths between HR practices and perceived internal and external employability, as well as the paths between perceived internal and external employability and workplace commitment were constrained to be equal showed no significant change in model fit (Δχ^2^ = -0.21, *p* = 0.98). An overview of the results of both samples is presented in Figure 1.

## Discussion

The central aim of our study was to apply a social exchange perspective to the HR practices – workplace commitment relationship and to explore the role of perceived employability as a potential mediator. Specifically, we hypothesized and found positive relationships between HR practices, perceived internal and external employability, and workplace commitment. We will next discuss our findings in more detail.

### Main Findings and Theoretical Implications

We showed that perceived employability mediates the positive relationship between the HR practices of training and development and commitment, and between participation and communication and commitment. This mediation was found for both perceived internal and external employability. These findings contribute to the literature in four ways.

First, our study contributes to the literature on HRM and employability (Van Harten et al., 2017). We showed that HR practices in terms of (1) training and development and (2) employee participation and communication relate to perceived internal and external employability, and workplace commitment in different ways. Allowing employees to participate in decision making and communicating clearly about processes and practices showed the strongest and most consistent positive relations with both internal and external perceptions of employability. Furthermore, training and development practices only related to internal employability perceptions, and performance feedback and reward did not relate to either form of perceived employability. These findings show that there is no overall relationship between HR practices and employability but that their interrelation depends on the type of HR practice that is studied. This is in line with the argument from Conway and Monks (2009) that HR has a rather complex effect on outcomes.

Much to our surprise, we did not establish a relationship between training and development practices and perceived external employability. This is in contrast to the idea that training in many cases leads to a formal accreditation that the employee can take to other organizations, and thus provide a signal to the external labor market. A possible explanation might be that employees attribute training programs primarily to their internal opportunities, as training practices are often related to enhancing job-related skills. It would be possible that their external marketability and employment opportunities are enhanced through training, yet that they do not see it as such, (i.e., their perceptions are not enhanced). Future research is needed to examine this relationship in more detail.

Another surprising finding in our study was that we found no support for the role of performance feedback and reward practices in enhancing commitment via employability. Looking more closely at the correlations, perceived internal employability and workplace commitment were both positively related to performance feedback and rewards in the 6-week follow-up sample, but none of those correlations was still significant in the 1-year follow-up sample. Furthermore, in the structural model, performance feedback and reward practices were not significantly related to commitment via employability. A first implication of these findings may be that receiving performance feedback and reward practices are specially valued in the short run but seems to disappear over time. The second implication is that even though there might be short-term advantages of performance feedback and rewards in terms of employability and commitment, these are apparently less important than participation and communication, and training and development. A possible explanation for this would be that performance reviews may not always be visible to employees, for example a monthly talk with a manager not being recognized as an actual HR practice (Beijer et al., 2019). It might also relate to the attributions that employees make, with training, development, participation, and communication practices being attributed to the organization in general, whereas performance feedback might predominantly trigger attributions toward to manager. Another possible explanation would be that performance appraisals and compensation practices may primarily trigger extrinsic motivation, which is more short-term in nature. Conversely, HR practices that help people develop (training and development) and be involved in their work (participation and communication) might trigger the need for competence, and thus more long-term intrinsic motivation (cf. Van den Broeck et al., 2016).

The second contribution is that our findings shed more light on the employability – commitment relationship. In line with our expectations, we found that both internal and external employability positively mediated the relationship between HR practices and workplace commitment. This contrasts the idea underlying the employability management paradox: at the core of the paradox is the idea that investing in employability is beneficial for organizations because it provides the organization with a competitive advantage in terms of human capital. Yet, it is risky when such investments also feed external employability, as it could pull employees out of the current employment relationship (De Cuyper and De Witte, 2011; Philippaers et al., 2017). Based on our results, we would say that the management paradox is highly tentative as we did not find any support for a negative relationship between external employability perceptions and workplace commitment. On the contrary: our findings indicate that there is a mutual win when organizations invest in employability enhancement as it strengthens employees’ knowledge and skills and also creates a committed workforce.

We should note that we found significant correlations for both employability and commitment with age. This correlation was negative for internal and external employability, and in both samples. These negative correlations are in line with most empirical findings on the relationship between age and employability (cf. Vanhercke et al., 2014) and could be explained as a consequence of employees changing in their goal orientation and future time perspective across the lifespan, as well as having fewer opportunities for learning once they get older (Froehlich et al., 2015). Age was positively correlated with commitment. Some prior studies have indeed found that commitment tends to increase with age (e.g., Allen and Meyer, 1993), and also that the type of HR practice that impacts commitment may change over one’s lifespan (Kooij et al., 2010). In all, these findings suggest that the role of employability enhancement in the HRM – commitment relationship may change over time and across career stages.

Third, we bring in a career perspective in the social exchange literature (Cropanzano and Mitchell, 2005). Perceived investments in HR practices relate to perceived internal employability, and this signals a mutual win and may therefore foster commitment: the win for employees is a career path within the organization and the win for employers is personnel with updated skills tailored to the organization’s needs. Likewise, those investments relate to perceived external employability: this implies a personal win for employees in the form of enhanced employment security beyond organizational boundaries, and they may want to secure this win by expressing commitment.

Fourth, our study contributes to existing HRM literature by putting employability forward as a mechanism in the HRM “black box,” that is, the unclear mechanisms through which HR practices are related to outcomes (Collins and Clark, 2003; Becker and Huselid, 2006). In our case, while the HR practices – commitment link has been firmly established in the literature, much less is known about *how* this process works. Based on our findings, perceptions of employability are a mechanism through which employer investments can lead to a more committed workforce.

### Limitations and Suggestions for Future Research

Our study has several limitations that need to be taken into consideration. First, although we had a large sample at T1 and the retention rate in the 6-week sample was quite high, (i.e., 43.6%), we ended up with a relatively small sample of 127 participants in the 1-year sample. The limited number of respondents who filled out the survey at all three measurement waves (89 employees) did not allow analyzing the three waves together. Although we could not test those three waves, we did replicate the results of the 6-week sample in the sample with a 1-year time lag, providing at least some cross validation of our findings. Another limitation related to the measurement at different times is that we could not control for earlier levels of perceived employability and commitment. Future studies could therefore use longitudinal panel designs to fully explore the causal and dynamic nature of employability.

A second limitation concerns the use of self-report measures. Although we followed the suggestions of Podsakoff et al. (2003), for example in using multiple measurement waves and performing statistical checks (e.g., CFA), the nature of our data might still have caused some common method variance. Yet, it can be argued that perceived employability and workplace commitment cannot be measured in other ways (Mäkikangas et al., 2004). Still, there are several avenues for future studies to strengthen our current findings. For example, by extending the research focus to include managerial reports of HR practices, future studies could also measure *actual* and *intended* HR practices (Ostroff and Bowen, 2016). Furthermore, future studies could also examine other, related outcomes such as sickness absence and productivity, to see whether employability might also be an explanatory mechanism between HRM practices and outcomes that can be more objectively measured.

As a suggestion for future research, we would encourage scholars to examine boundary conditions that might alter the relationships between employability and outcomes. Although we found no trade-offs of investing in employability, we acknowledge that there might be situations in which such investments could have unforeseen effects. For example, depending on the level of job enjoyment employees experience, the leadership they experience, or the level of actual opportunities for obtaining alternative employment, perceptions of employability might have a differential effect on outcomes. Consider someone who feels highly externally employable after receiving support from HRM and at the same time an external and upward job transition is offered, and/or that person does not get along with management very well, this might reduce commitment to the organization and potentially increase turnover. Future research should therefore further examine such boundary conditions to gain an even more complete understanding of the mechanisms associated with HRM investments in employability.

### Practical Implications

In line with recent studies (Nelissen et al., 2017; Philippaers et al., 2017), our findings indicate that HRM investments, when perceived by employees, can enhance perceived employability and, in turn, workplace commitment. The perception of being employable presents a win for the employee that is, at least in part, attributed to the organization, and thus creates commitment. Hence, it is important that organizations provide support in the development of their employees (cf. Benson, 2006) as this will induce reciprocity on their part. More specifically, we found that especially participation and communication practices were related to employability and commitment over time. Training and development had a short-term relationship with internal employability, whereas feedback and reward had no relationships with employability and commitment at all. These results indicate that organizations should focus on open communication and employee development in view of an employable and committed workforce.

These findings also call into question the discussion on the employability management paradox (De Cuyper and De Witte, 2011), which states that investments that lead to increased marketability might pull employees out of their current employment relationship. Our findings indicate that this is not the case. To the contrary, Those who perceived their employer’s investments were more likely to report higher levels of external employability, which was, in turn, positively related to workplace commitment, which can be explained by social exchange theory.

## Conclusion: Should Employers Invest in Employability?

Based on our results, we conclude that there are no clear risks for organizations when they use HR practices that can enhance employability. On the contrary, HR practices that relate to both internal and external employability are positively related to workplace commitment via social exchange processes. Therefore, our answer to the above question would be a clear “yes”: investing in employability seems to create a win-win scenario for employers and employees alike.

## Data Availability

The datasets generated for this study can be obtained from MT upon request.

## Author Contributions

JA performed the theorizing, analyzing, writing, and coordinating the research. MT performed the theorizing, analyzing, and writing the research. SB performed the theorizing, analyzing, and writing the research. NDC performed the theorizing and writing the research.

## Conflict of Interest Statement

The authors declare that the research was conducted in the absence of any commercial or financial relationships that could be construed as a potential conflict of interest.
